# Limited Dispersal Drives Strong Genetic Structure in the Commercially Harvested Gastropod *Buccinum undatum* in the Western North Atlantic

**DOI:** 10.1111/eva.70207

**Published:** 2026-02-02

**Authors:** Cassidy C. D'Aloia, Audrey Bourret, Krista D. Baker, Brigitte Desrosiers, Jonathan A. Kubelka, Claude Nozères, William H. Sturch, Geneviève J. Parent, Bruno L. Gianasi

**Affiliations:** ^1^ Department of Biology University of Toronto Mississauga Mississauga Ontario Canada; ^2^ Department of Ecology & Evolutionary Biology University of Toronto Toronto Ontario Canada; ^3^ Fisheries and Oceans Canada Maurice‐Lamontagne Institute Mont‐Joli Quebec Canada; ^4^ Fisheries and Oceans Canada Northwest Atlantic Fisheries Centre St. John's Newfoundland and Labrador Canada; ^5^ Department of Biological Sciences University of New Brunswick Saint John New Brunswick Canada

**Keywords:** conservation genomics, conservation units, direct‐developer, gastropod, seascape genomics

## Abstract

Direct‐developing species lack the pelagic larval phase which facilitates connectivity in most marine species. Consequently, they tend to exhibit spatially restricted dispersal and increased population structure. When subject to harvesting, this biological constraint increases their vulnerability to localized depletion, as local aggregations may be unable to recover through dispersal from neighboring areas. In eastern Canada, the direct‐developing whelk *Buccinum undatum* is targeted by commercial fisheries. Declining landings and catch per unit effort have raised concerns that the species' fully benthic life history renders it vulnerable to localized overexploitation. Here, we leverage a large genome‐wide dataset to elucidate patterns of spatial genetic structure in *B. undatum* and gain insight into how seascape features influence genetic connectivity. We sampled hundreds of individuals throughout Canadian northwest Atlantic waters and genotyped them at 23,405 SNPs. We detected five major genetic clusters, and considerable genetic substructure within most of these groupings. In the St. Lawrence Estuary, where geographic sampling was most intensive, isolation by distance, driven by limited dispersal along continuous habitat, was observed. Deep water also serves as a major barrier to gene flow, leading to genetic divergence among populations separated by less than 50 km. Exploratory analyses also indicate the potential for isolation by environment across the seascape. Overall, our results confirm the limited vagility and gene flow of *B. undatum* , which leads to hierarchical genetic structure across the seascape. These findings highlight the importance of managing whelk populations at local scales to protect distinct conservation units and support sustainable harvesting.

## 1 Introduction

Intraspecific genetic data underlie higher levels of biological diversity and have great potential to inform conservation and management strategies (Bernatchez et al. 2017; Andrello et al. 2023; Schmidt et al. 2023; Theissinger et al. 2023). Despite perceived challenges in integrating genetic and genomic data into conservation actions (Shafer et al. 2015; Taft et al. 2020), there is growing recognition of both the importance and feasibility of doing so. Indeed, the Convention on Biological Diversity's most recent Global Biodiversity Framework explicitly includes the protection of genetic diversity in its 2030 goals and targets (CBD 2022), and research is increasingly focusing on appropriate and feasible metrics. Examples include protecting genetic diversity within reserve networks, inferring connectivity rates and inbreeding levels, and estimating effective population size (Andrello et al. 2022; Nielsen et al. 2023; Hoban et al. 2022; Wilcox et al. 2023).

One of the most common applications of conservation genetics is the use of intraspecific genetic data to help define the spatial boundaries of populations and/or conservation units. Here, we define conservation units (CUs) in a broad sense, that is, as a general term that reflects discrete intraspecific units that conservation actions can be directed towards (see Moritz 1994; Fraser and Bernatchez 2001; Funk et al. 2012 for the nuances of defining CUs). Recent work demonstrates how spatial genetic patterns estimated with large SNP data sets can inform the designation of conservation units in terrestrial (Forester et al. 2022) and anadromous species (Xuereb et al. 2022; Lehnert et al. 2023) at a spatial resolution previously unavailable with smaller genetic data sets. More work is needed to define CUs using genomic data in marine species, which occupy an environment characterized by permeable barriers to gene flow (Selkoe et al. 2016).

One group of species particularly well‐suited to genetically guided CU designation is direct‐developing marine species. Direct developers, sometimes termed “aplanktonic,” have a fully benthic life history. Offspring develop within egg capsules on the seafloor and hatch as juveniles that resemble miniature adults, bypassing the free‐swimming larval stage common in most marine organisms (Pechenik 1999). Direct developers are analogous to many terrestrial animal species insofar as they spend their entire life cycle on the seafloor. Therefore, these species may be strongly influenced by benthic barriers to gene flow as opposed to pelagic flow patterns that are a dominant force influencing planktonic dispersers (White et al. 2010). Direct developers are typically expected to have more limited dispersal capacity than species with a planktonic larval stage (Hellberg 2009; Riginos et al. 2011), and thus may exhibit clearer population boundaries and stronger signals of reproductive isolation between populations.

The effect of larval developmental mode on spatial genetic structure has been well‐studied in several marine gastropod taxa. Notably, comparative work in littorinids has shown that species with direct development have stronger spatial population structure than congeners with pelagic larvae (Kyle and Boulding 2000; Lee and Boulding 2009; Blakeslee et al. 2021). Increased neutral genetic structure has been documented in other direct‐developing gastropod taxa (Collin 2001; Hoffman et al. 2011; López‐Márquez et al. 2021) and is also linked to restricted geographic ranges relative to pelagic dispersers (Barroso et al. 2022).

The waved whelk *Buccinum undatum* is a direct‐developing gastropod targeted by commercial fisheries throughout the North Atlantic Ocean. Despite its transatlantic distribution, the northwestern and northeastern lineages diverged approximately 2.1 million years ago and are, operationally, distinct units (Magnúsdóttir et al. 2019). This species lacks a planktonic larval phase, has internal fertilization (Martel et al. 1986), exhibits low overall mobility (Uboldi et al. 2025), and is attracted to baited traps (Himmelman 1988). Collectively, these traits are cited in stock assessments as representing localized overharvesting risks (e.g., Barrett 2023; Gianasi 2023). In Canada, *B. undatum* is currently managed within NAFO (Northwest Atlantic Fisheries Organization) subdivisions and is classified as a Major Fish Stock under the *Fisheries Act* with an “uncertain” stock status because it is data poor. Because it is understudied and has a unique life history compared to most major fish stocks, *B. undatum* is a good candidate for the application of genomic data to define CUs throughout the region where it is harvested, even though the species is not yet designated as “at risk” federally or provincially. Moreover, the Northwest Atlantic is a complex seascape, with strong variation in factors such as currents, temperature, salinity, and bathymetry (e.g., shallow shelf regions separated by deep channels). Genetic structure has already been documented in highly dispersive marine species in this seascape (Stanley et al. 2018; Lehnert et al. 2019; Dorant et al. 2022; Bourret et al. 2024), but the patterns and drivers of spatial genetic structure in dispersal‐limited species remain relatively understudied.

Preliminary research on *B. undatum* genetic patterns lays the groundwork for investigating connectivity across the Northwest Atlantic seascape. Several studies on the eastern Atlantic lineage have revealed genetic structure over small spatial scales (Pálsson et al. 2014; Goodall et al. 2021). Within the western Atlantic lineage, one study sampling *B. undatum* across nine locations throughout Atlantic Canada indicated that dispersal may be restricted across depth gradients and revealed a strong phylogeographic break off Nova Scotia on the Scotian Shelf, dividing the species into two distinct genetic clusters (Sturch and D'Aloia 2023). The first cluster spans the Bay of Fundy southward to Georges Bank, whereas the second extends from the central Scotian Shelf northward to waters surrounding the island of Newfoundland and the southern Gulf of St. Lawrence. Given the species' direct mode of development, further genetic subdivision across the seascape is expected. Moreover, expanding sampling efforts to underrepresented areas, including the St. Lawrence Estuary, could provide new insights into the genetic structuring of *B. undatum* across the Northwest Atlantic. To date, efforts to delineate hierarchical genetic subdivision have relied on relatively small SNP panels (~1000 SNPs; Sturch and D'Aloia 2023; Kubelka et al. 2025). Larger, genome‐wide SNP data with more power to disentangle spatial genetic patterns of *B. undatum* are required to assess how population genomics can support management goals.

Here, we address outstanding questions about *B. undatum* connectivity with a new data set comprised of hundreds of individuals sampled throughout Northwest Atlantic Canadian waters and genotyped at 23,405 SNPs. Specifically, we ask: (1) Is there genetic substructure that could inform the delineation of CUs at fishery‐relevant scales? (2) Do isolation‐by‐distance signals arise in continuously distributed populations given the species' direct mode of development? and (3) Is there evidence of isolation‐by‐environment? Our results indicate that *B. undatum* individuals are like hobbits (Palumbi and Warner 2003)—they stay close to home and do not disperse across deep water channels. This limited dispersal results in clearly defined genetic boundaries among populations and has important implications for the sustainable management of this commercially harvested marine resource.

## 2 Methods

### 2.1 Sample Collection and DNA Extractions

**FIGURE 1:**
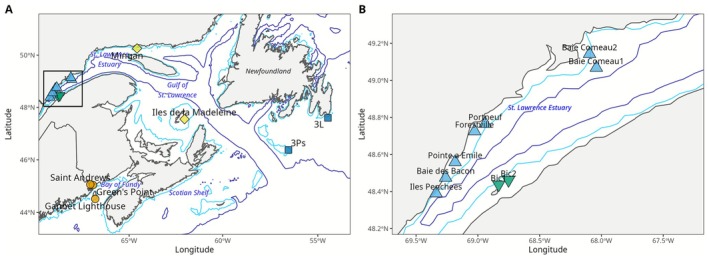
Sampling locations in five regions of the Northwest Atlantic (northern shore of St. Lawrence Lower Estuary in light blue triangles: NSLE; southern shore of St. Lawrence Lower Estuary in green inverted triangles: SSLE; Gulf of St. Lawrence in yellow diamonds: GSL; Bay of Fundy in orange circles; Southern Newfoundland in dark blue squares) (A); and zoom‐in on the St. Lawrence Lower Estuary where sampling sites are more closely spaced (B). Sampling sites are color‐coded by region. Isobath lines represent 50 (blue) and 250 (purple) m.

Individuals were collected from 16 locations during 2020–2023 using a combination of handpicking in the intertidal zone at low tides and trawls and baited traps in the subtidal zone (Table 1; Figure 1A). Immediately after collection, a small piece of foot tissue was preserved in 95% ethanol, with the ethanol swapped after 24 h. Samples from five sites were originally collected for previous population genetic studies of *B. undatum* (Sturch and D'Aloia 2023; Kubelka et al. 2025; Table 1). All individuals from the Estuary and Gulf of St. Lawrence were sexed before tissue preservation. DNA was extracted from all tissue samples, including the previously collected samples, using Qiagen DNeasy Blood & Tissue kits and quality checked on a 2% agarose gel. DNA concentrations were assessed on a Synergy LX plate reader (BioTek) using PicoGreen kits and normalized to a concentration of 10 ng/μL.

**TABLE 1:** Sampling sites with collection date, coordinates, and depth category, and the number of collected and retained samples after sequencing and filtering.

Region	Site	Depth	*N* sequenced	*N* retained	Latitude	Longitude	Date^a^	References
Bay of Fundy	Green's Point	Intertidal	33	32	45.04	−66.889	August 2020	Sturch and D'Aloia (2023)
Bay of Fundy	Saint Andrews	Intertidal	35	33	45.065	−67.037	September 2020	Sturch and D'Aloia (2023)
Bay of Fundy	Gannet Lighthouse	Subtidal	18	17	44.509	−66.779	March 2021	Sturch and D'Aloia (2023)
NSLE	Iles Penchees	Intertidal	35	24	48.3941	−69.3423	July 2023	
NSLE	Baie des Bacon	Intertidal	35	22	48.4791	−69.2659	July 2023	
NSLE	Pointe a Emile	Intertidal	35	28	48.5638	−69.1897	July 2023	
NSLE	Forestville	Subtidal	35	24	48.731	−69.0326	August 2022	
NSLE	Portneuf	Subtidal	35	35	48.7716	−68.9461	July 2023	
NSLE	Baie Comeau 1	Subtidal	35	35	49.0741	−68.032	August 2023	
NSLE	Baie Comeau 2	Subtidal	35	9^b^	49.1488	−68.0878	August 2022	
SSLE	Bic 1	Subtidal	35	35	48.4436	−68.8333	May 2023	
SSLE	Bic 2	Subtidal	35	34	48.4679	−68.7533	May 2023	
GSL	Mingan	Subtidal	35	24^c^	50.1757	−63.9665	April 2022	
GSL	Iles de la Madeleine	Subtidal	35	32	47.3263	−61.4456	July 2022	
Newfoundland	NAFO 3Ps	Subtidal	35	26^c^	45.5955	−55.7643	September 2022	Kubelka et al. (2025)
Newfoundland	NAFO 3L	Subtidal	35	21	46.488	−53.217	September 2020	Sturch and D'Aloia (2023)
		Total	541	431				

Abbreviations: GSL, Gulf of St. Lawrence; NSLE, northern Shore of St. Lawrence Lower Estuary; SSLE, southern Shore of St. Lawrence Lower Estuary.

^a^ We do not expect the sampling timespan (2020–2023) to impact results as *B. undatum* has a relatively long lifespan (~15 years; Brulotte 2019) and an advanced age at sexual maturity (~5–7 years in Canada; Gendron 1992; Ashfaq et al. 2019).

^b^ The retained individuals from Baie Comeau 2 were high quality, while the discarded were low quality.

^c^ Four individuals were removed from each of these sites as they were not *B. undatum* .

### 2.2 Library Preparation and ddRAD Sequencing

Six ddRAD libraries of 93–94 samples were prepared by the *Plateforme d'analyse génomique* (IBIS, Université Laval) using the restriction enzymes *pstI* and *mspI* following the protocol of Poland et al. (2012). In total, we sequenced 562 individuals, including 541 *B. undatum* (see Table 1), six technical replicates, five *Buccinum cyaneum* , six *Buccinum scalariforme* , and four *Buccinum terraenovae*. We included the congeners to test for species misidentification. All samples were sequenced on one lane of an Illumina NovaSeq 6000 with 150 bp, paired‐end reads at Génome Québec.

### 2.3 Bioinformatics and SNP Filtering

We performed initial read quality checks with fastQC (Andrews 2010) and multiQC (Ewels et al. 2016) and removed the universal Illumina adaptor with Trimmomatic (v0.39, Bolger et al. 2014). We demultiplexed reads using the *process_radtags* module of Stacks (v2.64, Catchen et al. 2013; Rochette et al. 2019), truncating reads to 90 base pairs and applying the default flags to clean, rescue, and quality check reads. To filter out any misidentified individuals, we first ran a *de novo* assembly in Stacks using all sequenced individuals. We used the *r80* method (Rochette and Catchen 2017) to identify appropriate parameters for a *de novo* assembly (*m* = 3; *M* = 4; *n* = 4) and built a catalog using individuals from all four *Buccinum* species. Ultimately, we removed four divergent individuals from Mingan, Quebec, which were neither *B. undatum* nor *B. cyaneum* , *B. scalariforme* , or *B. terraenovae*. Subsequent COI barcoding of three of these individuals revealed two were potentially *B. glaciale* and one was an unidentified *Buccinum* species.

Next, we repeated the *r80* method on just the putative *B. undatum* individuals to identify appropriate parameters for a species‐specific *de novo* assembly. Using 18 samples, we tested *m*: 3–5; *M*: 1–8; *n* = *M*−1, *M* + 1 and selected *m* = 3, *M* = 1, and *n* = 1. We ran the full Stacks pipeline using these parameters, assembling a catalog using 150 representative individuals with coverage ranging from 10–25×, which yielded a final catalog of 1,025,952 loci. All individuals with coverage > 5× (*n* = 535) were run through the gstacks module. Average effective per‐sample coverage was 16.2× (SD = 7.6×). Four additional individuals from NAFO subdivision 3Ps off the southern coast of Newfoundland showing suspiciously low percentages of reads retained by the pipeline were identified as *Colus* sp. based on shell reexamination and were removed from downstream analyses. We further only kept individuals with > 90,000 loci genotyped (*n* = 451), representing individuals with approximately > 8× coverage.

To filter the data (Table 2), we first used the populations Stacks module to retain loci with a minor allele frequency (MAF) of 0.01 present in 75% of all individuals. We then kept individuals with ≤ 30% and SNPs with ≤ 10% missing data. We removed loci with heterozygosity > 0.6 to remove any multilocus contigs and then filtered for excessively high or low depth coverage (< 10× or > 50× approximating the 1st and 99th quantile, respectively). Because we sequenced six libraries, we ran an RDA to identify any outlier SNPs attributable to batch effects. We also checked for sex‐linked loci by running an RDA with 150 known males and 150 known females and identifying outliers. We looked for individuals with suspiciously high kinship (*ϕ*) to remove any duplicate individuals using VCFtools (Danecek et al. 2011), but no pairs with *ϕ* > 0.3 were found. Finally, we retained only one SNP per locus (the first SNP) to reduce the likelihood of linkage disequilibrium and applied a more stringent MAF of 0.05. In total, we retained 431 individuals genotyped at 23,405 SNPs, with an overall missing data rate of 4.9%.

**TABLE 2:** Final SNP filtering steps after removing other species and individuals with low coverage. At each step, we list the number of individuals, loci, and SNPs retained.

Step(s)	Number individuals	Number loci	Number SNPs
(1) Loci present in 75% individuals; MAF = 0.01	451	101,645	161,785
(2) Retain individuals with ≤ 30% and SNPs with ≤ 10% missing data; remove technical replicates	431	48,636	100,393
(3) Remove loci Ho > 0.6	431	47,673	98,582
(4) Remove loci with low (< 10×; *n* = 39) and high (> 50×; *n* = 538) depth	431	47,214	97,819
(5) Filter outliers associated with library batch effect	431	46,828	96,095
(6) Filter sex‐linked loci	431	46,726	95,718
(7) Remove pairs with kinship > 0.3	431	46,726	95,718
(8) Keep one SNP per locus; MAF > 0.05	431	23,405	23,405

Finally, we identified outlier SNPs to test whether their exclusion impacted the detection of spatial genetic patterns, and whether outliers, on their own, yielded a different signal of genetic structure. We used PCadapt to identify outliers by retaining three PCs and calculating Bonferonni‐corrected *p*‐values (Privé et al. 2020). We identified *n* = 284 outliers and found that their exclusion had no impact on detecting population structure (Figure S1), and that, on their own, the outlier SNPs detected the same overarching signal of population structure (Figure S1). Therefore, the pattern between the “putatively neutral” and full SNP data set was congruent. We retained all 23,405 SNPs for most downstream analyses, adopting a total evidence approach.

### 2.4 Hierarchical Genomic Structure

To visualize genetic clustering of individuals, we conducted a principal component analysis (PCA) using all genotypes. We ran the *glPCA* function in *adegenet* (Jombart 2008), with alleles centered and 430 principal components retained. We color‐coded individuals by their sampling region and plotted ellipses around clusters. We considered five major geographic regions where our samples were collected: (1) the Bay of Fundy, (2) southern Newfoundland, (3) northern shore of the St. Lawrence Lower Estuary (NSLE), (4) southern shore of St. Lawrence Lower Estuary (SSLE), and (5) Gulf of St. Lawrence (GSL). To explore the effect of SNP panel size on genetic clustering and compare our results with prior studies based on modest SNP panels (Sturch and D'Aloia 2023; Kubelka et al. 2025), we randomly subsampled SNPs (*n* = 20,000, 10,000, 5000, 2000, 1000, 500, and 100 SNPs) and generated PCAs for each subsampled dataset.

Next, we assessed hierarchical patterns of genetic structure. We conducted a maximum likelihood mixed ancestry analysis in ADMIXTURE (v1.3.0, Alexander et al. 2009) and determined the optimal number of ancestral populations (*K*) with cross‐validation, testing from *K* = 1 to 20. Cross‐validation indicated several potential candidate values for *K*, and the PCA and prior population genetic analyses of *B. undatum* in the region (Sturch and D'Aloia 2023) both indicated the potential for strong hierarchical genetic structure within the Northwest Atlantic. Thus, we generated ADMIXTURE plots for increasing *K* values (*K* = 2, 3, 4, and 10). We also calculated population structure between all 16 sampling sites using the Weir and Cockerham (1984) pairwise *F*_ST_ estimate. We used the *gl.fst.pop* function in dartR and calculated 95% bootstrapped confidence intervals (*n* = 999 bootstraps). Pairwise *F*_ST_ values were visualized in a heatmap.

### 2.5 Self‐Assignment Tests

To test whether individuals assigned to the cluster they were sampled from, we conducted self‐assignment tests at two hierarchical levels using the *dapc* function in *adegenet*. First, we considered the five major genetic groups that are apparent in the first two PC axes (see Section 3). We applied 10‐fold cross validation to split our data into training and test sets, then assigned test individuals using the *predict* function. We repeated this 100 times to estimate accuracy. We also implemented this assignment analysis at a finer scale, considering the 10 subclusters that appear in subsequent PC axes and ADMIXTURE (see Section 3).

### 2.6 Evolutionary Relationships Among Populations

To gain insight into the evolutionary relationships among *B. undatum* from our 16 sampling sites and test for historical migration events, we used TREEMIX (v. 1.13) (Pickrell and Pritchard 2012). This method fits a bifurcating maximum likelihood tree to visualize the splits between populations and can also incorporate a series of historical migration events by adding additional edges. The tree was rooted using the three Bay of Fundy sites, identified as strongly genetically distinct from the other *B. undatum* (see Section 3). We did not use other *Buccinum* species as an outgroup because doing so would have drastically reduced the number of SNPs available for the analysis. We assumed SNP independence since we retained one SNP per locus. We added up to three historical migration events and assessed whether they improved the model fit to the data by comparing the amount of variance in genetic relatedness between sites explained by each model. We also used the three populations (f_3_) test to evaluate support for each potential historical migration event.

### 2.7 Exploring Broad‐Scale IBD and IBE Across the Seascape

At a broad spatial scale considering all sampled sites, we assessed the relative contribution of geographic (IBD) and environmental (IBE) variables to genetic variation using partial redundancy analyses (pRDA; Capblancq and Forester 2021). Latitude and longitude served as geographic explanatory variables. Environmental variables were selected from a broad pool of potential variables (mean annual/winter bottom and annual surface temperature, and mean annual salinity extracted from the BNAM model, Wang et al. 2018; depth) using both backward and forward selection procedures. The selected environmental variables were depth and annual mean bottom temperature. We emphasize that this analysis is exploratory in nature, as the modeled environmental variables have uncertainty for nearshore sites. To account for broad‐scale neutral genetic structure, we used the loadings of the first two axes of a PCA performed on putatively neutral SNPs (i.e., removing the PCadapt outliers). The full RDA model included the three sets of explanatory variables (geography, environment, neutral genetic structure), with minor allele frequency at each site as the response variable. Partial RDA was then used to partition the variance and quantify the independent effects of neutral genetic structure, IBD, and IBE.

### 2.8 Fine‐Scale Barriers to Gene Flow Within the St. Lawrence Lower Estuary

We tested several hypotheses related to how *B. undatum* 's limited dispersal capacity could impact emergent patterns of genetic structure in the seascape. First, we tested the hypothesis that limited dispersal capacity gives rise to genetic IBD along relatively linear habitats. Due to the patchy sampling design across the Northwest Atlantic and the broad spatial scale which included a known phylogeographic break, we restricted this analysis to the seven sites along the northern shore of the St. Lawrence Lower Estuary (NSLE region) where we had relatively continuous sampling (Figure 1B, blue triangles). We assessed whether linearized pairwise *F*_ST_ was correlated with logged in‐water geographic distance using a Mantel test (*n* = 999 permutations) and a linear model (Rousset 1997).

We also tested the hypothesis that depth differences (*z*) represent stronger barriers to gene flow than comparable latitudinal and longitudinal distances (*x*, *y*) in this species. We again focused on the St. Lawrence Estuary given the sparser sampling design in the rest of the study region (Figure 1A). However, in this analysis, we included comparisons between all sites on both the northern shore (NSLE; Figure 1B, blue triangles) and the two sites on the southern shore (SSLE; Figure 1B, green inverted triangles) which are separated by deep waters (> 250 m). We ran a linear model with linearized *F*_ST_ as the response variable and logged geographic distance, presence of a barrier (1/0), and their interaction as predictor variables. Here, a barrier represents whether a pair of sites is separated by the deep‐water channel running through the Estuary (1) or not (0). We also ran a partial Mantel test to assess whether the linearized *F*_ST_ matrix was correlated with logged in‐water geographic distance when accounting for the presence of a depth barrier between sites (*n* = 999 permutations).

## 3 Results

### 3.1 Hierarchical Genomic Structure

**FIGURE 2:**
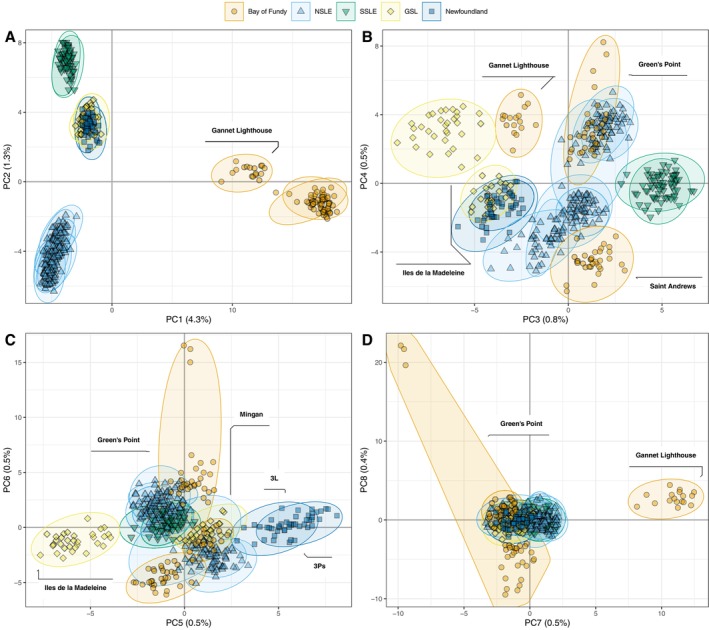
Principal component analysis of *B. undatum* genotyped at 23,405 SNPs. (A) PC1 and PC2; (B) PC3 and PC4; (C) PC5 and PC6; and (D) PC7 and PC8. Ellipses encompass individuals from the same site, with color representing the region.

The PCA performed over all 23,405 SNPs revealed hierarchical genomic clusters of *B. undatum* across the Northwest Atlantic. Broad‐scale patterns were identified, with the first two axes of the PCA revealing five distinct genetic clusters. The first two PC axes explained the largest proportion of variance, with 4.3% and 1.3%, respectively (Figure 2A). Two clusters of individuals from the Bay of Fundy sites (orange shading) were separated from all other sites along PC1 (Figure 2A). Within the Bay of Fundy sites, samples collected in (1) the offshore Gannet Lighthouse site were distinct genetically from those of (2) Green's Point and Saint Andrews, which are coastal sites. Along PC2, individuals were separated into three other major clusters: (3) the northern shore of the St. Lawrence Lower Estuary (NSLE, light blue triangles); (4) the southern shore of the St. Lawrence Lower Estuary (SSLE, green inverted triangles); and (5) the Gulf of St. Lawrence and Newfoundland sites (GSL, yellow diamonds; Newfoundland, blue squares). The latter cluster encompassed individuals from sites over a large geographic area, that is, from NAFO subdivision 3L off the southeastern coast of Newfoundland, up to the Northern shore of the Gulf of St. Lawrence at Mingan (Figure 1). These five major genetic clusters identified with the full 23,405 SNP data set were apparent when at least 5000 randomly selected SNPs were used (Figure S2).

Additional PCs revealed genetic substructure within some of these main clusters and explained variance ranging from 0.5% to 0.8% (Figure 2B–D). Specifically, PCs 3–4 (Figure 2B) and PCs 5–6 (Figure 2C) illustrated separation between samples from all three Bay of Fundy sites (orange shading); separation between Newfoundland sites (blue squares) and two of the Gulf of St. Lawrence sites (Îles de la Madeleine and Mingan, yellow diamonds); and some separation within the northern shore of the St. Lawrence Estuary (NSLE, light blue triangles).

**FIGURE 3:**
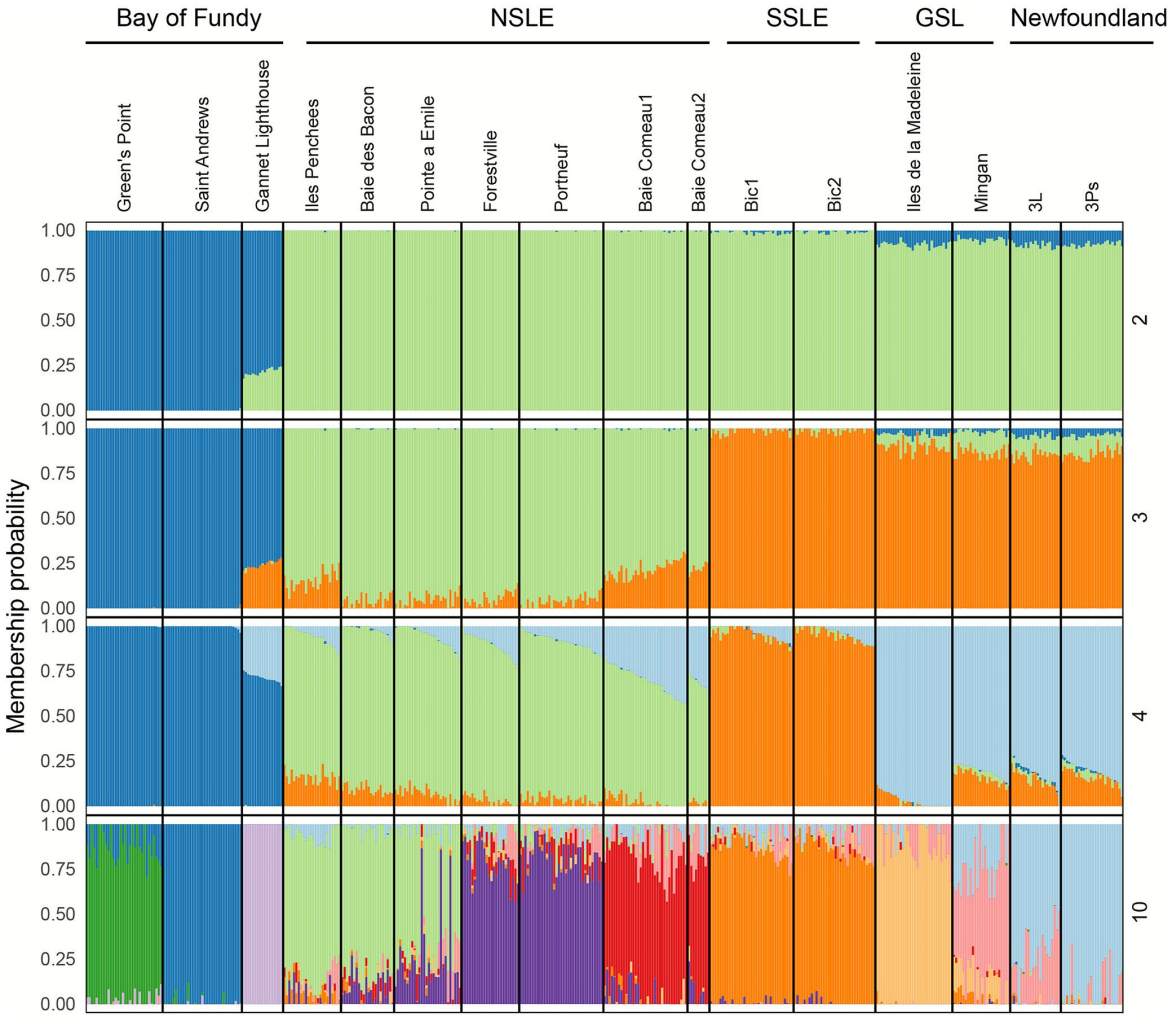
Hierarchical membership probability plots from ADMIXTURE analysis for *K* = 2, 3, 4, and 10 ancestral populations. Regional labels are above site names.

Admixture plots supported the PCA results showing hierarchical clustering (Figure 3). A plot of cross‐validation error at different *K* values revealed an elbow pattern (Figure S3), with strong support for *K* = 2–4. The broad scale patterns observed with the PCA in the first two PC axes were observed at *K* = 2–4. At *K* = 2, membership probabilities reflected the strong separation between the Bay of Fundy sites and the rest of the sites (Figure 3). At *K* = 3, individuals from the NSLE primarily assign to their own group, whereas at *K* = 4, individuals from the SSLE also assign to their own group.

Finer‐scale genetic differences within most genetic clusters identified by Admixture were revealed by plotting *K* = 10. We plotted *K* = 10 for several reasons: (1) strong hierarchical structure can mask subtler genetic structure at smaller spatial scales, (2) there was a steep increase in cross‐validation error between *K* = 10 and higher *K* values, (3) PC axes 3–7 indicated genetic substructure, and (4) all 10 clusters identified were geographically relevant. Indeed, within the Bay of Fundy, the three sites each formed distinct subclusters. The NSLE cluster was also separated into three subclusters which reflected the geographic distances between sites within this region and suggested an IBD pattern (Figure 1, see below for IBD test). Within the Newfoundland–Gulf of St. Lawrence genetic cluster, individuals from Mingan, Îles de la Madeleine, and Newfoundland each assigned to unique clusters (Figure 3; *K* = 10).

### 3.2 Self‐Assignment Tests

Self‐assignment accuracy to both the primary genetic groupings and the finer‐scale subclusters was very high. Considering the five main genetic clusters (Gannet Lighthouse, Coastal Bay of Fundy, GSL/Newfoundland, NSLE, and SSLE), the average self‐assignment percentage was 98.6% ± 0.01 SD. When we considered the *K* = 10 groups that were apparent in the finest‐scale ADMIXTURE analysis, we also observed a high mean self‐assignment accuracy of 88.1% ± 0.03 SD. Several subclusters had self‐assignment rates exceeding 90% (Figure S4). The subclusters with modest self‐assignments rates were those with more admixture with nearby clusters. For example, Mingan had 66% self‐assignment, with the remaining assignments going to Newfoundland and Îles de la Madeleine, which are subclusters it is admixed with (Figure 3; *K* = 10).

### 3.3 Pairwise *F*_ST_

**FIGURE 4:**
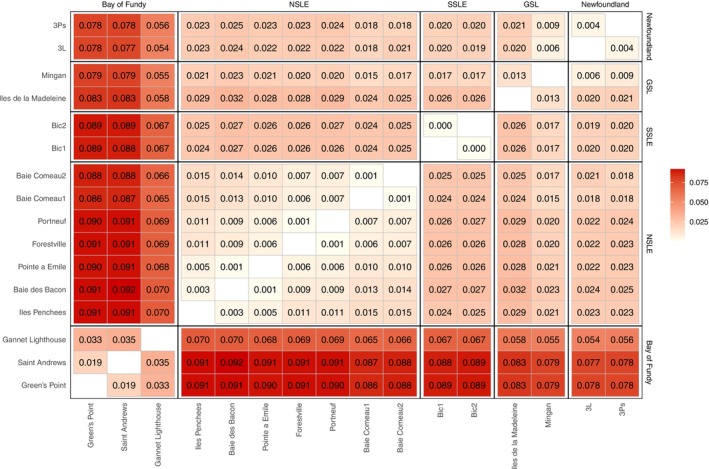
Symmetrical matrix of pairwise *F*_ST_ values between all sampling sites, grouped by region. Site labels are on the left and bottom; region labels are on the top and right. All values are statistically significant (*p* ≤ 0.024).

Average pairwise *F*_ST_ between sampling sites was 0.038, with a range of 0.0003–0.092 (all *p*‐values ≤ 0.024). The geographic patterns in the strength of structure were congruent with the PCA and Admixture results. For example, the strongest pairwise *F*_ST_ values were between the Bay of Fundy sites (Saint Andrews, Green's Point, Gannet Lighthouse) and all other sites (Figure 4, darker red shading), with values ranging from 0.054 to 0.092. Within the Bay of Fundy, there was also evidence of fairly strong structure between the geographically proximate sites Saint Andrews, Green's Point, Gannet Lighthouse (pairwise *F*_ST_ range 0.019–0.035) (Figure 4, upper left corner), corroborating the *K* = 10 admixture plot (Figure 3). Otherwise, pairwise values *within* all other regions were lower (*F*_ST_ ≤ 0.015).

### 3.4 Evolutionary Relationships Among Populations

**FIGURE 5:**
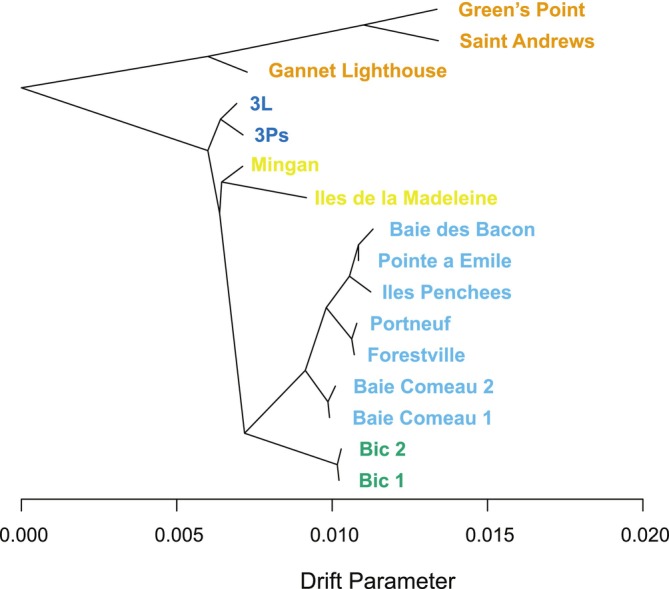
Evolutionary tree of *B. undatum* with no migration events recovered from TREEMIX. Residuals from the model are in Figure S5. Sampling site names are colored by region. Drift parameter reflects the amount of genetic drift that has occurred along the branch.

A bifurcating tree without historical migration events explained 99.4% of the variation in genetic relatedness among sampling sites (Figure 5), and its topology is consistent with the results of the population structure analyses. Rooting the tree with the genetically distinct Bay of Fundy region (Gannet Lighthouse, Saint Andrews, and Green's Point) revealed a first split between Newfoundland (3Ps and 3L) and the remaining Estuary and Gulf of St. Lawrence sites. A subsequent divergence separated the Mingan Archipelago in the northern Gulf and Îles de la Madeleine in the southern Gulf (GSL) from all sites in the St. Lawrence Lower Estuary. Finally, within the Lower Estuary, sites on the southern shore (SSLE) split from all sites on the northern shore (NSLE). In terms of historical mixture events, the residual plot indicated that a tree with additional edges might improve the fit for some sites (Îles de la Madeleine, Baie Comeau, and Îles Penchées; Figure S5). However, the increase in variance explained was marginal and, most importantly, none of the modeled migration events were supported by f_3_ statistics (Table S1).

### 3.5 IBE and IBD at Broad Spatial Scales

Considering all sampled sites, the partial RDA models partitioned total genetic variation into IBD, IBE, and neutral genetic structure (Table 3). The full model explained 70% of the total genetic variance (adjusted *R* ^2^ = 0.508, *p* = 0.001). Among the pRDA models, broad‐scale neutral genetic structure contributed the most, accounting for 16% of total variance (adjusted *R* ^2^ = 0.123, *p* = 0.001), followed by IBE with 11% (adjusted *R* ^2^ = 0.060, *p* = 0.002) and IBD with 9% (adjusted *R* ^2^ = 0.035, *p* = 0.012). A large proportion of variance was attributable to confounded effects among the three sets of variables, representing 35% of the total variance.

**TABLE 3:** Partial redundancy analysis (pRDA) partitioning the effect of neutral genetic structure, isolation by environment (IBE), and isolation by distance (IBD). The full model includes all explanatory variables, while neutral, IBE, and IBD models assess the effect of a set of explanatory variables while controlling for the others.

Partial RDA models	Inertia	Adjusted *R* ^2^	*p* (> *F*)	Prop. of explainable variance	Prop. of total variance
Full: F ~ neutral + env. + geo.	127.8	0.508	0.001	1.00	0.70
Neutral: F ~ neutral |(env. + geo.)	28.2	0.123	0.001	0.22	0.16
IBE: F ~ env. | (neutral + geo.)	19.9	0.060	0.002	0.16	0.11
IBD: F ~ geo. | (neutral + env.)	16.5	0.034	0.012	0.13	0.09
Confounded neutral/env./geo.	63.1			0.49	0.35
Total unexplained	53.6				0.30
Total variance	181.3				1.00

### 3.6 Fine‐Scale Barriers to Gene Flow Within the St. Lawrence Lower Estuary

**FIGURE 6:**
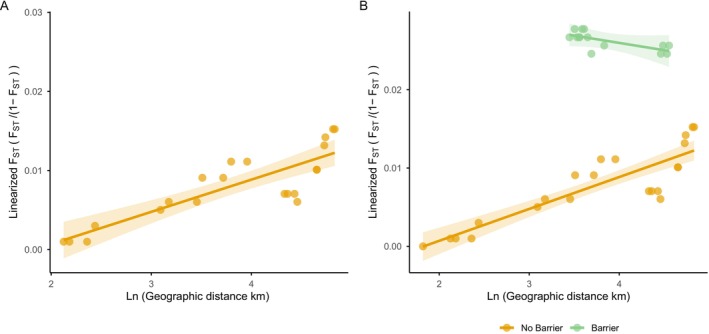
Patterns of isolation by distance (IBD) and isolation by deep water barrier within the St. Lawrence Estuary. (A) IBD for site pairs within NSLE only; (B) IBD for site pairs within NSLE or SSLE (orange points, *n* = +1 from A), but not for site pairs *across* the Estuary with a deep‐water barrier between them (green points). Points represent linearized *F*_ST_ values; lines and ribbons represent trendlines and 95% prediction intervals from linear models.

We next tested for fine‐scale distance and depth effects on genetic differentiation, focusing on the sites sampled within the St. Lawrence Lower Estuary. Considering only the northern shore (NSLE), where sampling occurred continuously along a single stretch of habitat, genetic IBD was confirmed (Mantel *r* = 0.85; *p* = 0.001) (Figure 6A). An accompanying linear model showed that logged geographic distance explained almost ¾ of the variance in linearized pairwise *F*_ST_ (*m* = 0.004; *t*‐value = 7.03; *p* < 0.001; *F*(1, 19) = 49.48; *R* ^2^ = 0.723).

We then expanded the geographic scope to include sites across the St. Lawrence Lower Estuary (i.e., to consider all pairwise comparisons both within and between NSLE and SSLE). Logged geographic distance was still a significant predictor of pairwise *F*_ST_ (*m* = 0.004; *t*‐value = 9.697; *p* < 0.001), as was the presence of a depth barrier (*m* = 0.041; *t*‐value = 8.175; *p* < 0.001), and the interaction between barrier presence and geographic distance (*m* = −0.006; *t*‐value = −4.601; *p* < 0.001) (Figure 6B). The interaction term revealed that when a depth barrier is absent, the slope between geographic distance and F_ST_ is positive (i.e., there is IBD—orange points, Figure 6B), but when a depth barrier is present (i.e., for pairs of sites that cross the Estuary), the relationship between geographic distance and *F*_ST_ disappears (green points, Figure 6B). Overall, the model explained a very high percentage of overall variation in linearized pairwise *F*_ST_, and this was largely attributable to the strong positive effect of a depth barrier on *F*_ST_ (*F*(3, 32) = 298.5; *p* < 0.001; adjusted *R* ^2^ = 0.962). A partial mantel test confirmed the correlation between geographic distance and linearized F_ST_ while controlling for the depth barrier matrix (Mantel *r* = 0.785; *p* = 0.006).

## 4 Discussion

Genomic data hold great promise to bolster the conservation and sustainable management of direct‐developing species. Because these species lack the pelagic larval phase that typically drives marine metapopulation connectivity, their limited dispersal capacity raises concerns about localized depletions when they are subject to overharvesting. Here, we address management‐relevant knowledge gaps related to genetic stock boundaries and connectivity in *Buccinum undatum* throughout the Canadian Atlantic. Drawing on genome‐wide SNPs, we uncovered remarkably clear signals of hierarchical genetic structure concordant with limited dispersal capacity, evidence that a deep‐water channel of at least 250 m is a strong barrier to gene flow, and preliminary evidence of IBE. Our results, when considered alongside regional variability in demography and declining catch per unit effort, indicate that small‐scale management areas with genomically informed targets of conservation units will be important to conserve *B. undatum* stocks.

### 4.1 Spatial Genetic Structure Across and Within Regions

We documented remarkably strong spatial genetic structure for a benthic marine species, with five major genetic clusters which could each be considered distinct conservation units (CUs). The five CUs are: (1) offshore Bay of Fundy; (2) coastal Bay of Fundy; (3) the northern shore of the St. Lawrence Lower Estuary (NSLE); (4) the southern shore of the St. Lawrence Lower Estuary (SSLE); and (5) the Gulf of St. Lawrence (GSL) and waters surrounding Newfoundland (Figure 2A). These major groupings contribute new information about how sites from the Estuary and northern shore of the Gulf of St. Lawrence fit within the Canadian whelk metapopulation, as these regions were unrepresented in a prior *B. undatum* population genetic study (Sturch and D'Aloia 2023). The strongest genetic split—that between the Bay of Fundy sites and the rest of the NW Atlantic sites—aligns with prior evidence of a phylogeographic split off Nova Scotia on the Scotian Shelf found in *B. undatum* (Sturch and D'Aloia 2023) and other species (Stanley et al. 2018). A potential simple explanation for the strong genetic difference is geographic isolation; the Bay of Fundy is separated from other sampled populations by approximately 950–1700 km in‐water distances and whelks have limited capacity to disperse far distances given their direct mode of development. An alternative explanation is that Canadian shores could have been colonized from two distinct refugia following the last glacial maximum (Maggs et al. 2008). From a southern refugium, populations could have recolonized northward into the Bay of Fundy. From an Atlantic Canadian refugium around Newfoundland/Grand Banks, populations could have recolonized westward into the Estuary and Gulf of St. Lawrence.

Concordant with expectations for a dispersal‐limited direct developer, we also observed genetic subdivision within most of these primary clusters, suggesting that smaller‐scale CUs are present in parts of the study region. This finding was most apparent in the Admixture analyses wherein individuals from specific sampling locations assigned to unique ancestral populations. This was true within the Bay of Fundy, where each site was assigned to a unique ancestral population, and to a lesser extent in the St. Lawrence Lower Estuary (see green, purple, and red bars in *K* = 10 plot, Figure 3). These fine‐scale genetic signals using 23,405 SNPs confirm patterns that were hinted at in prior work using modest SNP panels (Sturch and D'Aloia 2023; Kubelka et al. 2025), underscoring the value of large marker panels in elucidating subtle genetic structure in marine species (Alves Monteiro et al. 2024; Bourret et al. 2024; Babaei et al. 2025).

Genetic substructure was weak across the Gulf of St. Lawrence and Grand Banks (southeast of Newfoundland) but was still apparent. Whelks from Mingan and from Îles de la Madeleine, occurring across hundreds of kilometers and the Laurentian Channel, were subtly distinct from each other and also from those of Newfoundland (Grand Banks). In contrast, whelks from the two NAFO subdivisions off southern Newfoundland, which are not divided by a deepwater channel, were genetically similar. Previous work has suggested that within Newfoundland waters, relatively homogeneous habitats at similar depth profiles could facilitate intergenerational stepping‐stone dispersal (Kubelka et al. 2025) and our results support this logic.

Overall, these spatial genetic patterns were supported by the inferred evolutionary relationships between populations, with TREEMIX revealing a set of population splits that mirrors the PCA and Admixture groupings. Notably, the strong fit of the bifurcating tree *without* major historical mixture events aligns with expectations for a species that lacks pelagic dispersal. We postulate that the positive residuals at a few sites (Figure S4), which were not resolved by adding more migration edges, could instead be explained by genetic structure between our sampled sites and unsampled sites. Given the broad geographic scale of our study region and the fact that we observed strong structure in the intensively sampled subregion of the St. Lawrence Lower Estuary, there are likely to be additional genetic groupings. For example, whelks from unsampled sites on the Scotian Shelf and along the southern portion of the GSL are likely to have shared drift with Îles de la Madeleine, which had the poorest model fit.

### 4.2 Limited Dispersal as a Driver of Isolation by Distance in Continuous Habitat

Given that *B. undatum* exhibits strong signals of genetic structure and lacks a pelagic larval phase, assessing whether IBD contributes to population structure within the western Atlantic whelk lineage was the next question to be resolved. IBD has been documented in various marine species with pelagic larval phases (Benestan et al. 2021; Naaykens and D'Aloia 2022; Macleod et al. 2024) and in the eastern Atlantic *B. undatum* lineage throughout the southern North Sea, English Channel, and Irish sea (Morrissey et al. 2022). Here, we document a clear IBD signal in a species without pelagic larval dispersal along the northern shore of the St. Lawrence Lower Estuary. Because IBD patterns at the population level are a function of genetic drift and migration (Malécot 1948; Rousset 1997; Wright 1943), we infer that this pattern is influenced by spatially restricted dispersal along this continuous, relatively linear one‐dimensional habitat.

Although more continuous sampling throughout the species range would likely detect IBD along other coastlines, we do not expect IBD to arise across all parts of the seascape. Indeed, a partial RDA indicated only a modest effect of IBD on genetic variation across all sampled sites. We note, however, that this was a conservative test of IBD since broad‐scale neutral genetic structure was also accounted for. Considering another region, Kubelka et al. (2025) found no evidence of IBD within a relatively homogenous seafloor inside NAFO subdivision 3Ps off the southern coast of Newfoundland. This finding could be explained by more potential gene flow routes within the two‐dimensional 3Ps habitat compared to the approximately one‐dimensional habitat along the northern shore of the St. Lawrence Lower Estuary. Alternatively, seascape barriers to gene flow may overpower subtler IBD signals. In our study, this pattern is seen within the Estuary itself, where the IBD signal erodes for pairs of sites on either side of a deep‐water channel (Figure 6B—green line). Nevertheless, in documenting a clear IBD signal for the first time, our work complements recent telemetry work that uncovers spatially restricted movements of whelks over time (Uboldi et al. 2025). We conclude that both demographic and genetic connectivity are highly spatially restricted in northwest Atlantic *B. undatum* populations in Canadian waters.

### 4.3 Depth as a Strong Barrier to Gene Flow and the Potential for IBE

Our data support the hypothesis that depth influences patterns of genetic structure, an idea that has been highlighted in previous studies on both sides of the Atlantic for this species (Weetman et al. 2006; Mariani et al. 2012; Goodall et al. 2021; Sturch and D'Aloia 2023) and other marine invertebrates (Serrano et al. 2014; Prada and Hellberg 2021; Sturm et al. 2023). Our primary test of this hypothesis comes from the St. Lawrence Lower Estuary. Whelks occurring on either side of the Lower Estuary are separated by a deep‐water channel (> 250 m) and their average pairwise *F*_ST_ is three times higher than sites occurring on the same side of the Estuary. Although we do not know the history of colonization into both sides of the Estuary following the last glacial maximum, the deep‐water channel acts as a contemporary barrier to gene flow. The Estuary was the only region where we had a sufficient sample size to statistically test this hypothesis, but we observed a similar trend in the Bay of Fundy. There, an increase in pairwise *F*_ST_ coincides with the presence of deep water (> 150 m) between the two coastal sites (St. Andrew's and Green's Point) and Gannet Lighthouse.

In seascape genomics, the effect of depth may be interpreted as a proxy for unmeasured environmental covariates (e.g., temperature, substrate, and light or food availability), but here we suggest that depth per se may serve as a barrier to gene flow given the species' direct mode of development. If *B. undatum* populations are not abundant in deep channels, genetic structure may arise over time if few individuals traverse far vertical distances. Although the species has been described as having a broad depth distribution (Macpherson 1971), surveys indicate that whelk densities peak at relatively shallow subtidal depths (e.g., 20 m in North Sea (Valentinsson et al. 1999); 50–60 m in Mid Atlantic Bight (Borsetti et al. 2018)). In the Gulf of St. Lawrence, the fishery is concentrated in shallow coastal waters and very few individuals are captured on trawls deeper than 100 m (Chamberland et al. 2025). Telemetry data from this region also reveal that individuals tagged in shallow waters stayed within a narrow depth profile (~10–30 m) (Uboldi et al. 2025). Thus, we conclude that for this fully benthic species, strong depth differences along the seafloor, combined with decreased population densities in deep waters, reduce the likelihood of movement and gene flow across major depth zones.

Although the focus of this study was assessing neutral genetic structure, we also document preliminary, modest evidence of IBE (where environment is represented by depth and annual mean bottom temperature). We caution that this IBE result should *not* be considered for management purposes, as the modeled environmental variables are at a coarse resolution and were developed for the subtidal environment only. Instead, this preliminary result sets the stage for future studies that can more rigorously test for evidence of local adaptation by coupling genomic data from more sampling sites with better estimates of local environmental conditions and shell phenotypes. This result also aligns with IBE and local adaptation evidence from other direct‐developing gastropods. In *Littorina saxatilis* , for example, genetically divergent ecotypes have distinct phenotypes associated with intertidal microhabitats (Grahame et al. 2006; Le Pennec et al. 2017; Kess et al. 2018). Future studies characterizing adaptive genetic variation in *B. undatum* can also be integrated into the designation of CUs (Xuereb et al. 2022; Lehnert et al. 2023).

### 4.4 Management Implications

In Canada, whelks are managed within specific fishing zones nested inside broader NAFO (Northwest Atlantic Fisheries Organization) subdivisions. Our results support the relevance of this small‐scale management approach. In Quebec alone, there are 15 designated whelk fishing areas, and we detected substantial genetic structure across this region, including within the Estuary itself. Moreover, recently published telemetry data from the Gulf of St. Lawrence showed that individual whelks exhibited limited movement, with net displacement of < 1 km over 1–2 years (Uboldi et al. 2025). This restricted mobility is consistent with our findings of fine‐scale genetic differentiation (Figures 2, 3, 4), suggesting that gene flow among fishing areas is also minimal. Although not originally designed from a genetic perspective, fishing areas may nonetheless function as effective proxies for local CUs.

Overall, our data reinforce prior concerns that *B. undatum* 's fully benthic life history increases its vulnerability to localized overexploitation. Complementary demographic and landings data show spatial variability in size at sexual maturity (Ashfaq et al. 2019; Gianasi 2023; Kubelka et al. 2025) and declining CPUE (Gianasi 2023). Given the limited potential for demographic and genetic rescue effects, management measures such as license allocations, quotas, and/or size limits should be carefully evaluated within fishing zones alongside genetic data to ensure stock sustainability. Furthermore, if new fisheries are to be developed, our results suggest it is fundamental to consider the degree of geographic isolation and the existence of any nearby depth barriers, as these are strong indicators of genetically distinct and potentially more vulnerable whelk populations.

## Funding

This work was supported by the Harrison McCain Foundation (HMF2019 YS‐04); Fisheries and Oceans Canada (SFSF‐2022‐NL03); New Brunswick Innovation Foundation (CEF2021‐026, RIF2019‐034); Natural Sciences and Engineering Research Council of Canada (RGPIN‐2020‐04112); The National Integrated Marine Response Planning Program ‐ DFO.

## Ethics Statement

Samples from the Bay of Fundy were collected under DFO Scientific License # 360846 to C.C.D. All other samples were collected by DFO staff as part of ongoing surveys and did not require a license.

## Conflicts of Interest

The authors declare no conflicts of interest.

## Supporting information


**Figure S1:** Principal component analysis of *B. undatum* genotyped at (A, B) putatively neutral and (C) outlier SNPs, and (D) Manhattan plot, with outliers indicated in orange.
**Figure S2:** PCAs (axes 1 and 2) of all *B. undatum* individuals at decreasing numbers of randomly selected SNPs.
**Figure S3:** (A) Percent variation explained by each axis in the PCA. (B) Cross‐validation of number of potential ancestral populations (*K*) from ADMIXTURE.
**Figure S4:** Heatmaps of self‐assignment percentages from 100 repetitions of cross‐fold validation assignments.
**Figure S5:** Supporting TREEMIX output.

**Table S1:** Output from f3 tests conducted in TREEMIX, sorted from lowest to highest Z‐score.

## Data Availability

Data and code for this study are available at: https://github.com/GenomicsMLI‐DFO/Buccin_ddRAD_2024.
