# Supplementary material for: Limited Dispersal Drives Strong Genetic Structure in the Commercially Harvested Gastropod Buccinum undatum in the Western North Atlantic
Source: Evol Appl. 2026 Feb 2;19(2):e70207. doi: 10.1111/eva.70207 (PMC12865132; doi:10.1111/eva.70207)
Supplement: Supplementary file 1 — Figure S1: Principal component analysis of B. undatum genotyped at (A, B) putatively neutral and (C) outlier SNPs, and (D) Manhattan plot, with outliers indicated in orange. Figure S2: PCAs (axes 1 and 2) of all B. undatum individuals at decreasing numbers of randomly selected SNPs. Figure S3: (A) Percent variation explained by each axis in the PCA. (B) Cross‐validation of number of potential ancestral populations (K) from ADMIXTURE. Figure S4: Heatmaps of self‐assignment percentages from 100 repetitions of cross‐fold validation assignments. Figure S5: Supporting TREEMIX output. [file EVA-19-e70207-s001.docx]

**Limited dispersal drives strong genetic structure in the commercially harvested gastropod *Buccinum undatum* in the Western North Atlantic**

**Supplementary Figures**


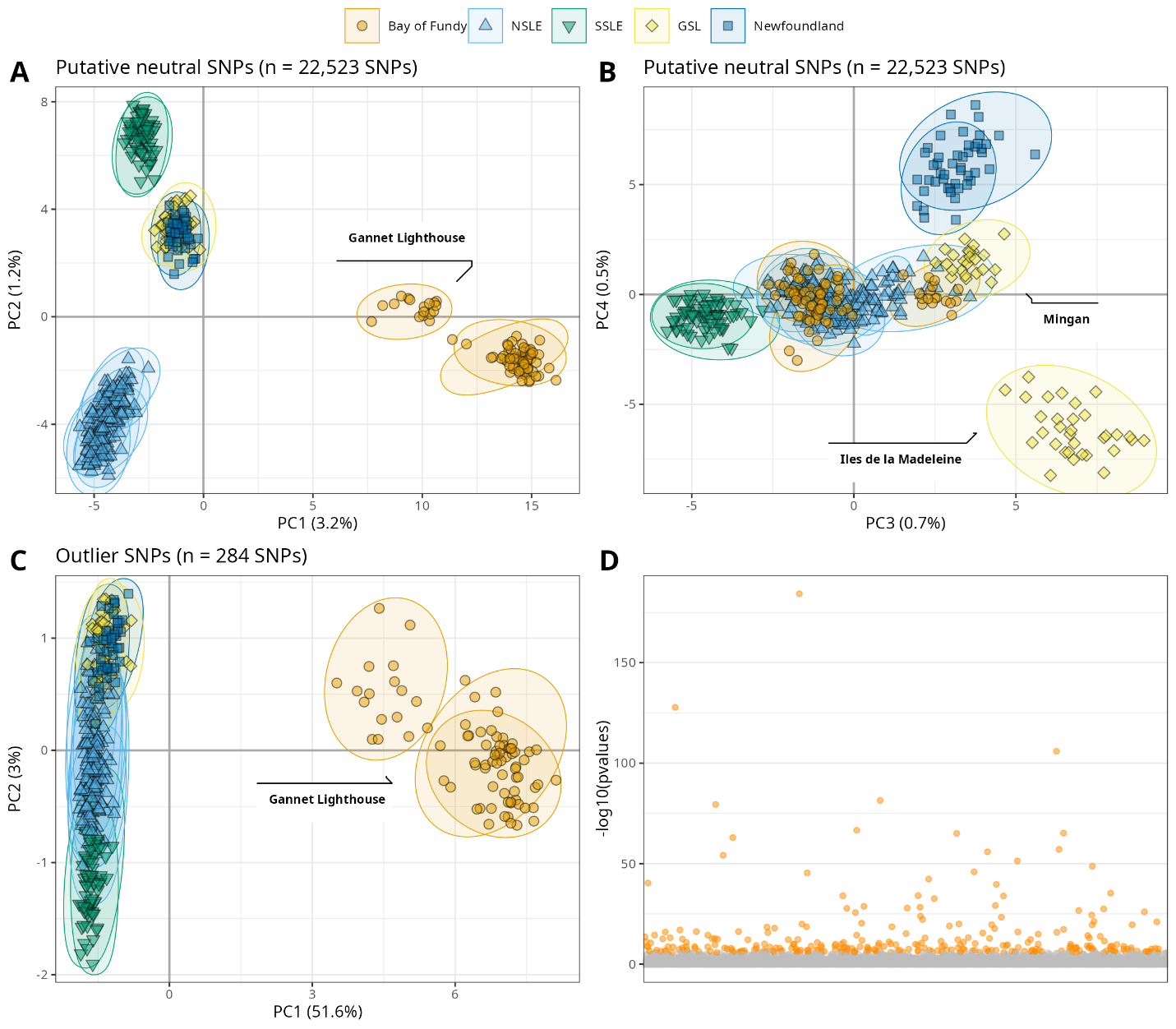


**Figure S1**. Principal component analysis of *B. undatum* genotyped at (**A-B**) putatively neutral and (**C**) outlier SNPs, and (**D**) Manhattan plot, with outliers indicated in orange. Excluding the outliers (*n* = 862 without Bonferroni correction) reveals the same genetic clusters as the full data set, while using only outliers (*n* = 284 with Bonferroni correction) primarily divides Bay of Fundy individuals from the rest along PC1 (51.6% variation explained).


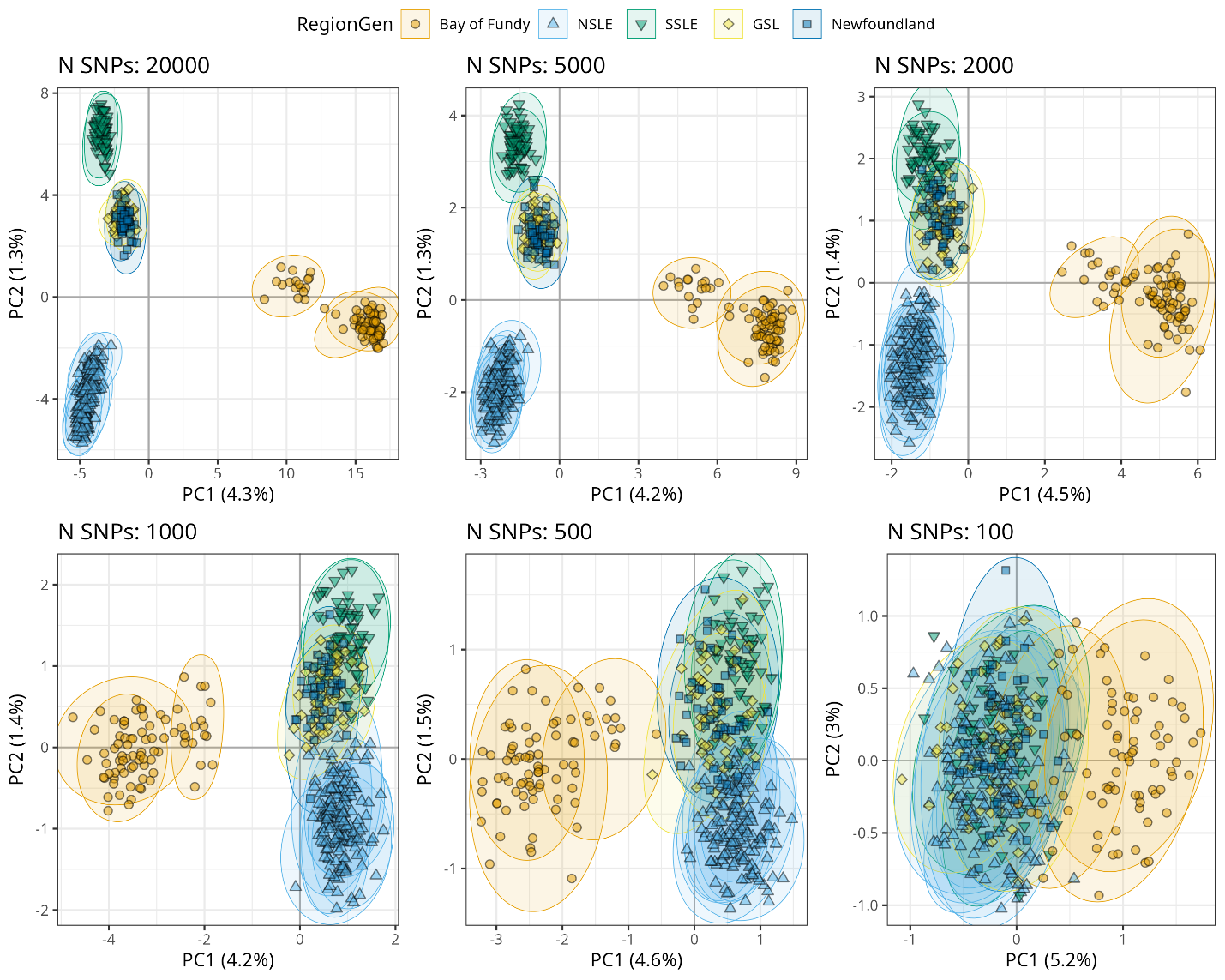


**Figure S2**. PCAs (axes 1 and 2) of all *B. undatum* individuals at decreasing numbers of randomly selected SNPs.

**
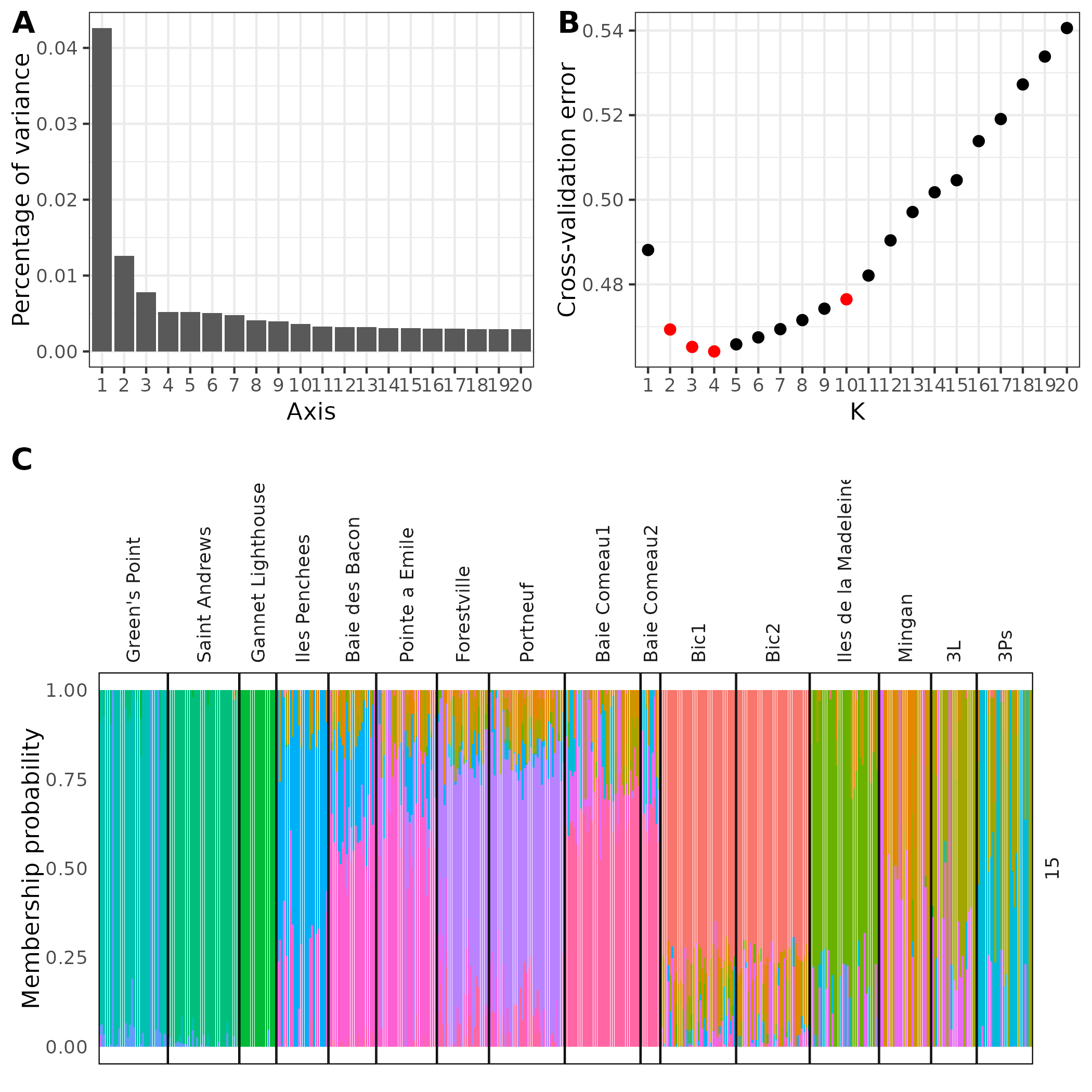
**

**Figure S3**. **A** Percent variation explained by each axis in the PCA. **B** Cross-validation of number of potential ancestral populations (K) from ADMIXTURE. To explore hierarchical structure, in the main text we generated admixture plots for the red values (i.e., values leading to the lowest cross-validation error (K = 2-4) as well as a higher value (K = 10) to test for real substructure which could be obscured by stronger patterns at broad spatial scales.


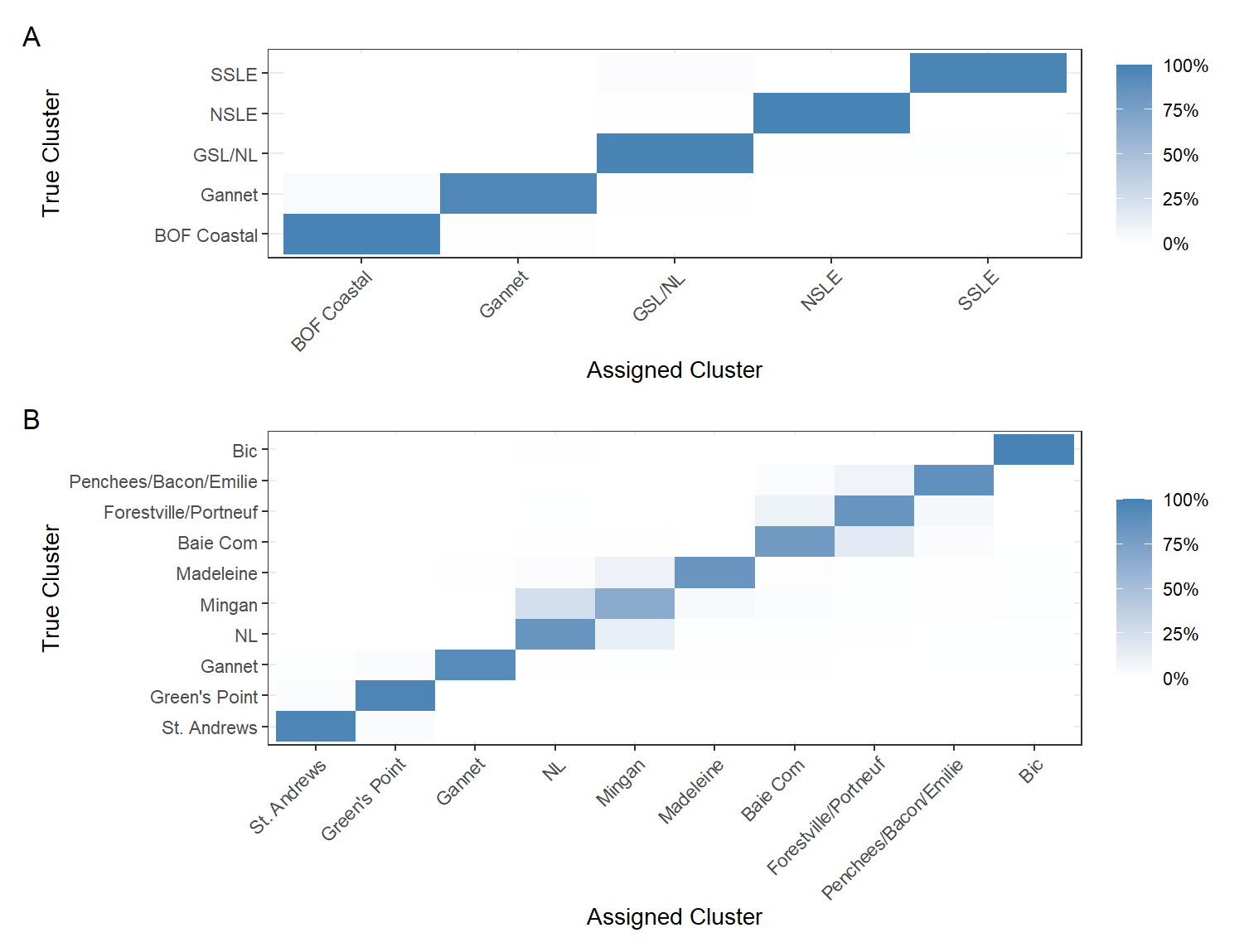


**Figure S4**. Heatmaps of self-assignment percentages from 100 repetitions of cross-fold validation assignments. **A** Assignments to the five major clusters; **B** Assignment to the 10 subclusters associated with the K=10 ADMIXTURE analysis. Self-assignments are along the diagonals.


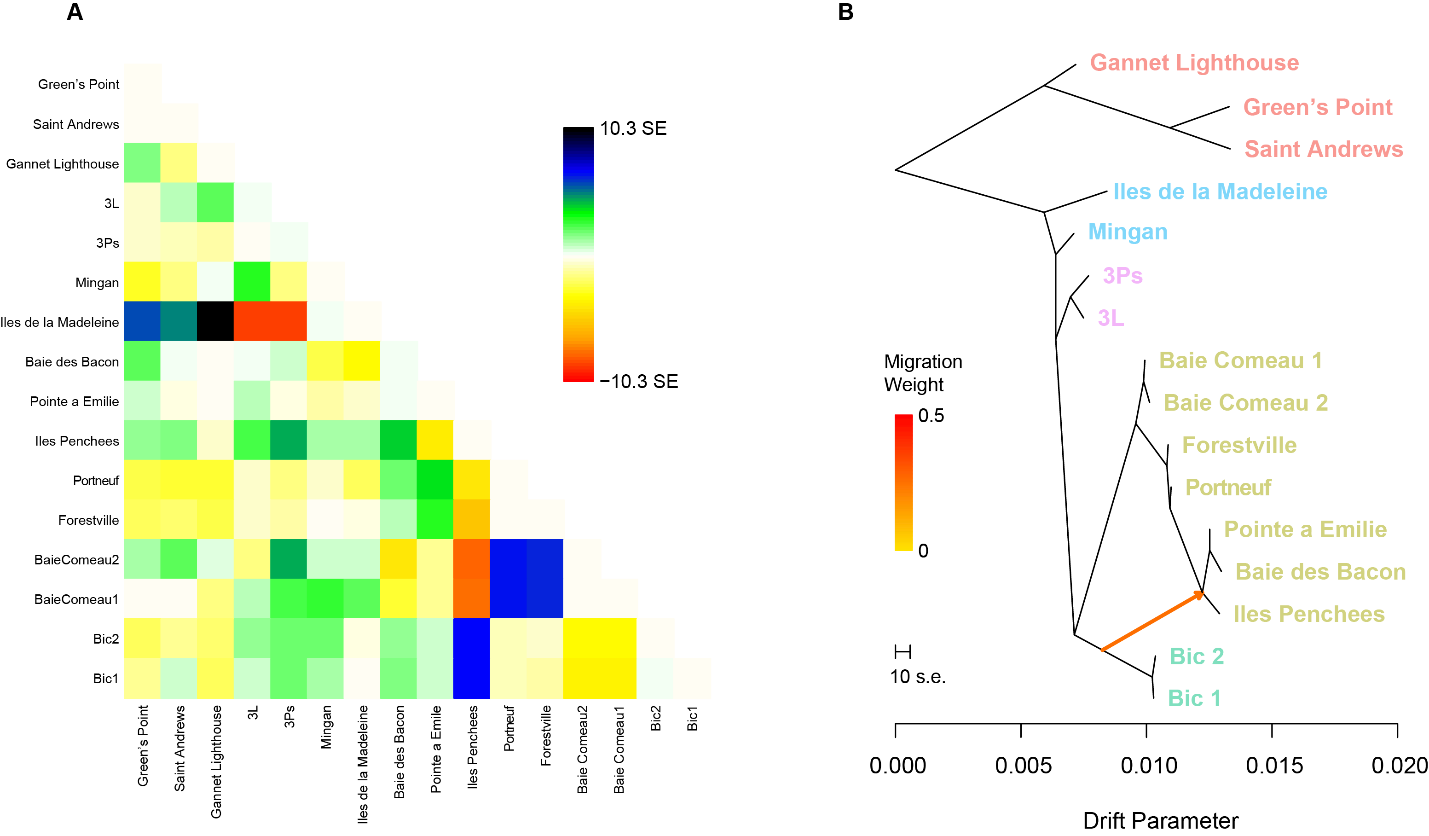


**Figure S5**. Supporting TREEMIX output. **A** Residuals from TREEMIX model without migration. **B** Example tree with first potential migration event explains 99.6% of the variance in population relatedness. However, this mixture event, along with others tested, was not supported by f3 statistics, so we retain the model with only population splits in the main text (see Table S1).
